# Editorial

**Published:** 1875-04

**Authors:** 


### Editorial.

### THE SAME OLD STORY.

We have recently been repeatedly informed that the agents of the Dental Vulcanite Company, are notifying dentists that the use of celluloid for artificial dentures is an infringement of their patents for making dental plates of rubber! And that prosecutions will be commenced against all who dare to use it. Now so far as we are able to learn, the vulcanite company have no more claim upon celluloid or its use for dental purposes than they have upon anything else that does not belong to them. We doubt not they would demand license for the use of gold for dental purposes even to that of filling teeth, if there was a ghost of a hope that thier claim might be sustained.

As long as members of the profession will consent to pay money to this company to satisfy what all dentists regard as unjust claims, just so long will they make their claims and issue their threats of prosecution, execution and imprisonment. Dr. S. S. White who is the agent for the sale of celluloid, says, "I am not aware that Bacon has obtained a "controlling" or any other interest in the celluloid base, and as I am the sole agent for its sale I am sure such a thing could not happen without my knowledge." And now to all we say abandon rubber, and use celluoid. We are receiving evidences almost every day of the adaptability of celluloid for dental purposes. The following from Dr. W. P. Hall, is similar to many others. He says, "I have been using celluloid with great satisfaction to myself and patients in more than an hundred cases, and without a single case failing thus far. If it shall prove durable in the mouth, retaining its integrity and color, there can be no doubt of its superiority to rubber, and will ere long entirely supersede it."

Dr. H. has large experience, and ventures his opinion and statement only after mature deliberation and experiment. The success of a half dozen reliable men with any mode or process, is worth more than the failures of a thousand. Let all try it.

### COMMENCEMENT.

The Commencement exercises of the twenty-ninth annual session of the Ohio College of Dental Surgery was held on the evening of Thursday, March 4th, in the College building. A large number of the profession and friends of the institution were present. The exercises were those common upon such occasions. The degrees were conferred by Dr. James Taylor, President of the Board of Trustees, upon the following gentlemen, viz:

W. A, Spaulding, Minneapolis, Minn.
A. M. Callaham, Topeka, Kansas.
D. S. Dibble, Ashland, Ky.
C. E. Case, Cincinnati, Ohio.
C. B. Mower, Wooster, Ohio.
F. W. Stewart, Sidney, Ohio.

The annual address was delivered by Dr. Joseph Richardson, of Terre Haute, Ind., and the valedictory on behalf of class was delivered by Dr. W. A. Spaulding. Thus ended one of the most pleasant and satisfactory session ever held in this institution. A spring course will be held, beginning on Monday, April 5th, and continuing till the first of June.

The next annual session will begin on the 11th of October next.

Honorary degrees were conferred upon J. S. Rice, of Shelbyville, Ind., and H. M. Reid, of Cincinnati, Ohio.

### THE OHIO DENTAL COLLEGE ASSOCIATION

Held its annual meeting on the 2d of March, 1875. The usual business was transacted, showing the financial affairs of the college to be in a very satisfactory condition.

The Board of Trustees consists of the following gentlemen: Jas. Taylor, President; H. A. Smith, Secretary; Jas. Leslie, J. G. Cameron, B. D. Wheeler, H. R. Smith, of Cincinnati; W. H. Morgan, of Nashville, Tenn.; Geo. W. Keely, Oxford, Ohio; H. J. McKillop, St. Louis, Mo.

### THE MISSISSIPPI VALLEY DENTAL SOCIETY

Held its thirtieth annual session March 3d, 4th and 5th. There was a very full attendance—larger than for several years before. Quite a number of interesting and important subjects were presented and discussed. Several very good papers were read, which will appear in the pages of the Register. A full report of the discussions is being prepared for publication. New methods of operating and modes of practice were fully considered, and many new and valuable ideas and suggestions were put forth.

There was a considerable increase in the membership.

This Society undoubtedly has a long career of usefulness before it yet. Long may it live and work.

